# Bis[μ-bis­(diphenyl­phosphino)methane-κ^2^ *P*:*P*′]bis­[(chloro­difluoro­acetato-κ*O*)silver(I)](*Ag*—*Ag*)

**DOI:** 10.1107/S1600536808011033

**Published:** 2008-04-26

**Authors:** Kong Mun Lo, Seik Weng Ng

**Affiliations:** a Department of Chemistry, University of Malaya, 50603 Kuala Lumpur, Malaysia

## Abstract

The asymmetric unit of the title compound, [Ag_2_(C_2_ClF_2_O_2_)_2_(C_25_H_22_P_2_)_2_], consists of two half-mol­ecules, each Ag^I^ ion lying on a center of symmetry. In each complete mol­ecule, two bis­(diphenyl­phosphino)methane ligands bridge two Ag^I^ ions, which are further coordinated by one chloro­difluoro­acetate ligand, giving *T*-shaped geometries and short intra­molecular Ag⋯Ag distances of 3.1078 (6) and 2.9950 (6) Å. In one mol­ecule, the unique –CF_2_Cl group is rotationally disordered over two sites with approximate occupancies of 0.53 and 0.47 for the major and minor components, respectively.

## Related literature

The compound is isostructural with [Ag(O_2_CCF_3_)(C_6_H_5_)_2_PCH_2_P(C_6_H_5_)_2_]_2_, see: Effendy *et al.* (2005 ▶). The report also provides background literature on complexes of bis­(di­phenyl­phosphino)methane with univalent coinage metals.
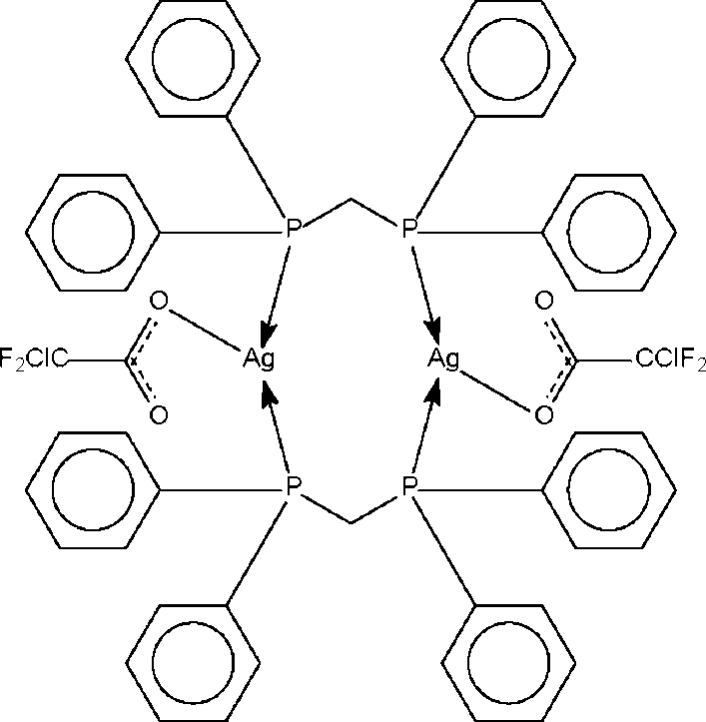


## Experimental

### 

#### Crystal data

[Ag_2_(C_2_ClF_2_O_2_)_2_(C_25_H_22_P_2_)_2_]
*M* *_r_* = 1243.41
Triclinic, 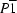

*a* = 10.8127 (3) Å
*b* = 14.4911 (3) Å
*c* = 17.2340 (3) Å
α = 83.040 (1)°
β = 86.477 (1)°
γ = 72.188 (1)°
*V* = 2551.1 (1) Å^3^
*Z* = 2
Mo *K*α radiation
μ = 1.06 mm^−1^
*T* = 100 (2) K
0.30 × 0.20 × 0.15 mm

#### Data collection

Bruker SMART APEXdiffractometer
Absorption correction: multi-scan (*SADABS*; Sheldrick, 1996 ▶) *T*_min_ = 0.648, *T*_max_ = 0.857
31942 measured reflections
11567 independent reflections
8338 reflections with *I* > 2σ(*I*)
*R*_int_ = 0.066

#### Refinement

*R*[*F* ^2^ > 2σ(*F* ^2^)] = 0.045
*wR*(*F* ^2^) = 0.112
*S* = 1.01
11567 reflections
641 parameters
54 restraints
H-atom parameters constrained
Δρ_max_ = 0.91 e Å^−3^
Δρ_min_ = −1.05 e Å^−3^

### 

Data collection: *APEX2* (Bruker, 2007 ▶); cell refinement: *SAINT* (Bruker, 2007 ▶); data reduction: *SAINT*; program(s) used to solve structure: *SHELXS97* (Sheldrick, 2008 ▶); program(s) used to refine structure: *SHELXL97* (Sheldrick, 2008 ▶); molecular graphics: *X-SEED* (Barbour, 2001 ▶); software used to prepare material for publication: *publCIF* (Westrip, 2008 ▶).

## Supplementary Material

Crystal structure: contains datablocks global, I. DOI: 10.1107/S1600536808011033/lh2610sup1.cif

Structure factors: contains datablocks I. DOI: 10.1107/S1600536808011033/lh2610Isup2.hkl

Additional supplementary materials:  crystallographic information; 3D view; checkCIF report

## Figures and Tables

**Table:** 

Ag1—O1	2.382 (3)
Ag1—P1	2.4194 (12)
Ag1—P2^i^	2.419 (1)
Ag2—O3	2.383 (3)
Ag2—P3	2.430 (1)
Ag2—P4^ii^	2.429 (1)

**Table:** 

O1—Ag1—P1	100.92 (9)
O1—Ag1—P2^i^	103.78 (9)
P1—Ag1—P2^i^	151.19 (4)
O3—Ag2—P3	104.14 (8)
O3—Ag2—P4^ii^	101.43 (8)
P3—Ag2—P4^ii^	149.67 (4)

Symmetry codes: (i) 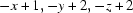; (ii) 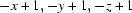.

**Fig. 1.**
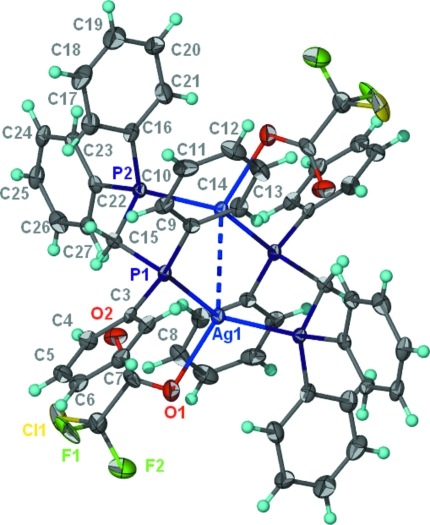
70% Probability thermal ellipsoid plot of one independent formula unit of (I). Hydrogen atoms are drawn as spheres of arbitrary radius. The disorder is not shown.

**Fig. 2.**
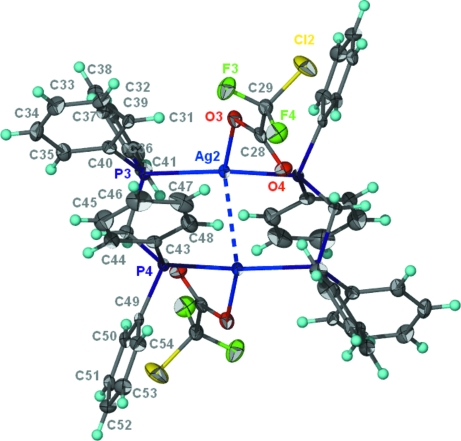
70% Probability thermal ellipsoid plot of the ither independent formula unit of (I). Hydrogen atoms are drawn as spheres of arbitrary radius.

## supplementary crystallographic information

### Comment

A recent report on complexes of bis(diphenylphosphino)methane with univalent coinage metals details the structure of the 1:1 adduct of the ligand with silver trifluoroacetate. The dinuclear molecule has the ligand binding to two carboxylate-bound silver atoms, who geometry is best described as being *T*-shaped (Effendy *et al.*, 2005). Replacing the trifluoroacetate ion by the chlorodifluoroacetate ion gives the isostructural title compound (I). There are two formula units in the unit cell (Figs. 1 and 2). One of the unique chlorodifluromethyl groups is ordered whereas the other is disordered.

### Experimental

Silver chlorodifluoroacetate was prepared *in situ* from silver oxide (1.20 g, 5 mmol) and chlorodifluoroacetic acid (0.65 g, 5 mmol) in acetone (50 ml). The reactants were heated until the silver oxide dissolved completely. To the brown solution was added bis(diphenylphosphino)methane (3.84 g, 10 mmol) dissolved in acetone (50 ml). The mixture was heated for 30 min; the colorless filtered solution yielded prism-shaped crystals in 60% yield.

### Refinement

Carbon-bound H-atoms were placed in calculated positions (C—H 0.95 to 0.99 Å) and were included in the refinement in the riding model approximation, with *U*(H) set to 1.2*U*_eq_(C).

There is minor disorder in the chlorodifluoromethyl groups. For both the ordered and disordered groups, the C–Cl distances were restrained to within 0.01 Å of each other, as were the C–F distances. The Cl···F distances as well as the F···F distances were similarly restrained. The Cl1 atom is near the F1' atom, the Cl1' atom near the F1 atom and the F2 atom near the F2' atom; for these pairs, the temperature factors were restrained to be the same. The ansiotropic temperature factors of the Cl1, F1 and F2 atoms were restrained to be nearly isotropic.

The largest peak and deepest holes area in the vicinity of the diordered chlorodifluoromethyl group.

### Crystal data

**Table:**

[Ag_2_(C_2_ClF_2_O_2_)_2_(C_25_H_22_P_2_)_2_]	*Z* = 2
*M**_r_* = 1243.41	*F*_000_ = 1248
Triclinic, *P*1	*D*_x_ = 1.619 Mg m^−^^3^
Hall symbol: -P 1	Mo *K*α radiation λ = 0.71073 Å
*a* = 10.8127 (3) Å	Cell parameters from 4611 reflections
*b* = 14.4911 (3) Å	θ = 2.3–25.1º
*c* = 17.2340 (3) Å	µ = 1.06 mm^−^^1^
α = 83.040 (1)º	*T* = 100 (2) K
β = 86.477 (1)º	Block, colorless
γ = 72.188 (1)º	0.30 × 0.20 × 0.15 mm
*V* = 2551.1 (1) Å^3^	

### Data collection

**Table:**

Bruker SMART APEXII diffractometer	11567 independent reflections
Radiation source: fine-focus sealed tube	8338 reflections with *I* > 2σ(*I*)
Monochromator: graphite	*R*_int_ = 0.066
*T* = 100(2) K	θ_max_ = 27.5º
ω scans	θ_min_ = 1.2º
Absorption correction: multi-scan(SADABS; Sheldrick, 1996)	*h* = −14→11
*T*_min_ = 0.648, *T*_max_ = 0.857	*k* = −18→18
31942 measured reflections	*l* = −22→22

### Refinement

**Table:**

Refinement on *F*^2^	Secondary atom site location: difference Fourier map
Least-squares matrix: full	Hydrogen site location: inferred from neighbouring sites
*R*[*F*^2^ > 2σ(*F*^2^)] = 0.045	H-atom parameters constrained
*wR*(*F*^2^) = 0.112	*w* = 1/[σ^2^(*F*_o_^2^) + (0.0475*P*)^2^ + 0.1513*P*] where *P* = (*F*_o_^2^ + 2*F*_c_^2^)/3
*S* = 1.01	(Δ/σ)_max_ = 0.001
11567 reflections	Δρ_max_ = 0.91 e Å^−^^3^
641 parameters	Δρ_min_ = −1.05 e Å^−^^3^
54 restraints	Extinction correction: none
Primary atom site location: structure-invariant direct methods	

### Fractional atomic coordinates and isotropic or equivalent isotropic displacement parameters (Å^2^)

**Table:**

	*x*	*y*	*z*	*U*_iso_*/*U*_eq_	Occ. (<1)
Ag1	0.57141 (3)	0.89080 (2)	1.025045 (18)	0.01359 (9)	
Ag2	0.44457 (3)	0.57795 (2)	0.435262 (17)	0.01308 (9)	
P1	0.36794 (11)	0.90512 (8)	1.09660 (6)	0.0128 (2)	
P2	0.21898 (11)	1.08082 (8)	0.99185 (6)	0.0126 (2)	
P3	0.22156 (11)	0.57580 (8)	0.45928 (6)	0.0123 (2)	
P4	0.33747 (11)	0.49679 (8)	0.61526 (6)	0.0123 (2)	
Cl1	0.4662 (5)	0.6779 (3)	0.79374 (16)	0.0304 (8)	0.528 (4)
Cl1'	0.4581 (6)	0.5832 (3)	0.9291 (2)	0.0379 (9)	0.472 (4)
Cl2	0.57724 (14)	0.91381 (9)	0.36753 (7)	0.0349 (3)	
F1	0.4587 (11)	0.6087 (7)	0.9329 (5)	0.0379 (9)	0.528 (4)
F2	0.6495 (6)	0.5949 (9)	0.8867 (7)	0.057 (3)	0.528 (4)
F1'	0.4795 (11)	0.6915 (7)	0.8078 (4)	0.0304 (8)	0.47
F2'	0.6572 (6)	0.6182 (9)	0.8661 (8)	0.057 (3)	0.47
F3	0.3672 (3)	0.93038 (18)	0.44623 (16)	0.0303 (7)	
F4	0.5391 (3)	0.89416 (18)	0.51371 (14)	0.0281 (7)	
O1	0.6050 (3)	0.7406 (2)	0.97176 (19)	0.0252 (8)	
O2	0.4233 (3)	0.8250 (2)	0.90831 (18)	0.0250 (8)	
O3	0.4269 (3)	0.7468 (2)	0.40995 (17)	0.0194 (7)	
O4	0.5974 (3)	0.7093 (2)	0.48919 (17)	0.0210 (7)	
C1	0.5177 (5)	0.7528 (3)	0.9251 (3)	0.0177 (10)	
C2	0.5279 (4)	0.6620 (3)	0.8834 (2)	0.0320 (13)	
C3	0.3471 (4)	0.7884 (3)	1.1364 (2)	0.0134 (9)	
C4	0.3135 (5)	0.7311 (3)	1.0871 (2)	0.0182 (10)	
H4	0.2984	0.7544	1.0335	0.022*	
C5	0.3020 (5)	0.6412 (3)	1.1149 (3)	0.0203 (10)	
H5	0.2785	0.6030	1.0807	0.024*	
C6	0.3245 (4)	0.6058 (3)	1.1930 (3)	0.0195 (10)	
H6	0.3159	0.5438	1.2125	0.023*	
C7	0.3599 (4)	0.6621 (3)	1.2422 (3)	0.0192 (10)	
H7	0.3757	0.6381	1.2956	0.023*	
C8	0.3725 (4)	0.7528 (3)	1.2146 (2)	0.0176 (10)	
H8	0.3981	0.7903	1.2486	0.021*	
C9	0.3251 (4)	0.9819 (3)	1.1755 (2)	0.0151 (9)	
C10	0.2197 (5)	0.9807 (3)	1.2262 (2)	0.0196 (10)	
H10	0.1697	0.9385	1.2203	0.023*	
C11	0.1883 (5)	1.0417 (3)	1.2856 (3)	0.0247 (11)	
H11	0.1177	1.0401	1.3208	0.030*	
C12	0.2601 (5)	1.1050 (3)	1.2934 (3)	0.0284 (12)	
H12	0.2387	1.1464	1.3341	0.034*	
C13	0.3617 (5)	1.1072 (3)	1.2422 (3)	0.0250 (11)	
H13	0.4094	1.1515	1.2467	0.030*	
C14	0.3950 (5)	1.0457 (3)	1.1842 (3)	0.0197 (10)	
H14	0.4666	1.0470	1.1497	0.024*	
C15	0.2313 (4)	0.9546 (3)	1.0303 (2)	0.0147 (9)	
H15A	0.1496	0.9527	1.0588	0.018*	
H15B	0.2440	0.9141	0.9865	0.018*	
C16	0.0933 (4)	1.1592 (3)	1.0494 (2)	0.0161 (9)	
C17	−0.0165 (5)	1.1347 (3)	1.0790 (2)	0.0213 (10)	
H17	−0.0218	1.0712	1.0751	0.026*	
C18	−0.1181 (5)	1.2016 (4)	1.1138 (3)	0.0282 (12)	
H18	−0.1916	1.1837	1.1352	0.034*	
C19	−0.1112 (5)	1.2954 (4)	1.1172 (3)	0.0331 (13)	
H19	−0.1813	1.3419	1.1402	0.040*	
C20	−0.0046 (5)	1.3213 (4)	1.0877 (3)	0.0312 (13)	
H20	−0.0015	1.3857	1.0901	0.037*	
C21	0.1000 (5)	1.2531 (3)	1.0541 (3)	0.0257 (11)	
H21	0.1749	1.2706	1.0347	0.031*	
C22	0.1465 (4)	1.0963 (3)	0.8966 (2)	0.0136 (9)	
C23	0.0346 (4)	1.1716 (3)	0.8754 (2)	0.0153 (9)	
H23	−0.0120	1.2131	0.9129	0.018*	
C24	−0.0092 (5)	1.1862 (3)	0.7991 (2)	0.0192 (10)	
H24	−0.0854	1.2377	0.7847	0.023*	
C25	0.0581 (5)	1.1261 (3)	0.7447 (2)	0.0201 (10)	
H25	0.0284	1.1363	0.6927	0.024*	
C26	0.1690 (5)	1.0508 (3)	0.7656 (3)	0.0228 (11)	
H26	0.2148	1.0092	0.7279	0.027*	
C27	0.2132 (5)	1.0358 (3)	0.8403 (2)	0.0175 (10)	
H27	0.2896	0.9841	0.8540	0.021*	
C28	0.5066 (4)	0.7659 (3)	0.4504 (2)	0.0159 (9)	
C29	0.4938 (4)	0.8749 (3)	0.4479 (2)	0.0180 (10)	
C30	0.0954 (4)	0.6894 (3)	0.4722 (2)	0.0147 (9)	
C31	0.1256 (4)	0.7772 (3)	0.4582 (2)	0.0150 (9)	
H31	0.2112	0.7774	0.4423	0.018*	
C32	0.0285 (5)	0.8645 (3)	0.4677 (2)	0.0186 (10)	
H32	0.0485	0.9244	0.4587	0.022*	
C33	−0.0963 (5)	0.8647 (3)	0.4902 (3)	0.0225 (11)	
H33	−0.1615	0.9248	0.4963	0.027*	
C34	−0.1273 (5)	0.7780 (3)	0.5038 (3)	0.0230 (11)	
H34	−0.2133	0.7782	0.5191	0.028*	
C35	−0.0303 (5)	0.6904 (3)	0.4948 (2)	0.0189 (10)	
H35	−0.0507	0.6307	0.5043	0.023*	
C36	0.1645 (4)	0.5244 (3)	0.3820 (2)	0.0129 (9)	
C37	0.0671 (4)	0.5790 (3)	0.3314 (2)	0.0156 (9)	
H37	0.0206	0.6443	0.3399	0.019*	
C38	0.0372 (5)	0.5388 (4)	0.2684 (3)	0.0244 (11)	
H38	−0.0290	0.5767	0.2337	0.029*	
C39	0.1038 (5)	0.4439 (3)	0.2564 (3)	0.0231 (11)	
H39	0.0833	0.4165	0.2133	0.028*	
C40	0.1998 (5)	0.3883 (3)	0.3063 (3)	0.0231 (11)	
H40	0.2447	0.3227	0.2981	0.028*	
C41	0.2302 (5)	0.4287 (3)	0.3684 (3)	0.0199 (10)	
H41	0.2972	0.3905	0.4024	0.024*	
C42	0.2131 (4)	0.4938 (3)	0.5487 (2)	0.0137 (9)	
H42A	0.1259	0.5155	0.5740	0.016*	
H42B	0.2286	0.4266	0.5354	0.016*	
C43	0.2848 (4)	0.6229 (3)	0.6363 (2)	0.0134 (9)	
C44	0.1606 (5)	0.6641 (3)	0.6664 (2)	0.0200 (10)	
H44	0.1048	0.6248	0.6792	0.024*	
C45	0.1174 (5)	0.7612 (3)	0.6780 (3)	0.0274 (12)	
H45	0.0329	0.7884	0.6995	0.033*	
C46	0.1990 (6)	0.8198 (3)	0.6579 (3)	0.0313 (13)	
H46	0.1688	0.8873	0.6644	0.038*	
C47	0.3228 (6)	0.7794 (3)	0.6289 (3)	0.0306 (13)	
H47	0.3781	0.8191	0.6159	0.037*	
C48	0.3675 (5)	0.6805 (3)	0.6185 (3)	0.0219 (10)	
H48	0.4536	0.6526	0.5994	0.026*	
C49	0.3078 (4)	0.4276 (3)	0.7060 (2)	0.0132 (9)	
C50	0.2893 (4)	0.3372 (3)	0.7047 (2)	0.0159 (9)	
H50	0.2852	0.3139	0.6560	0.019*	
C51	0.2767 (4)	0.2804 (3)	0.7737 (3)	0.0182 (10)	
H51	0.2634	0.2189	0.7722	0.022*	
C52	0.2837 (5)	0.3134 (3)	0.8447 (3)	0.0220 (10)	
H52	0.2737	0.2750	0.8920	0.026*	
C53	0.3050 (5)	0.4020 (3)	0.8470 (3)	0.0220 (10)	
H53	0.3121	0.4237	0.8959	0.026*	
C54	0.3161 (5)	0.4597 (3)	0.7781 (2)	0.0175 (10)	
H54	0.3295	0.5212	0.7800	0.021*	

### Atomic displacement parameters (Å^2^)

**Table:**

	*U*^11^	*U*^22^	*U*^33^	*U*^12^	*U*^13^	*U*^23^
Ag1	0.01193 (18)	0.01480 (16)	0.01485 (16)	−0.00570 (13)	0.00048 (13)	−0.00078 (12)
Ag2	0.01034 (18)	0.01539 (16)	0.01353 (16)	−0.00444 (13)	0.00060 (13)	−0.00059 (12)
P1	0.0113 (6)	0.0147 (5)	0.0135 (5)	−0.0060 (5)	0.0002 (4)	−0.0003 (4)
P2	0.0111 (6)	0.0137 (5)	0.0135 (5)	−0.0050 (5)	0.0003 (4)	−0.0001 (4)
P3	0.0109 (6)	0.0130 (5)	0.0129 (5)	−0.0037 (5)	−0.0005 (4)	−0.0009 (4)
P4	0.0109 (6)	0.0134 (5)	0.0130 (5)	−0.0049 (4)	0.0006 (4)	−0.0003 (4)
Cl1	0.0465 (17)	0.0385 (15)	0.0165 (13)	−0.0238 (11)	−0.0117 (11)	−0.0080 (11)
Cl1'	0.0584 (16)	0.025 (2)	0.0436 (13)	−0.0322 (17)	0.0145 (11)	−0.0131 (13)
Cl2	0.0471 (9)	0.0215 (6)	0.0367 (7)	−0.0159 (6)	0.0141 (6)	0.0016 (5)
F1	0.0584 (16)	0.025 (2)	0.0436 (13)	−0.0322 (17)	0.0145 (11)	−0.0131 (13)
F2	0.038 (3)	0.042 (5)	0.091 (6)	0.000 (2)	0.001 (3)	−0.041 (4)
F1'	0.0465 (17)	0.0385 (15)	0.0165 (13)	−0.0238 (11)	−0.0117 (11)	−0.0080 (11)
F2'	0.038 (3)	0.042 (5)	0.091 (6)	0.000 (2)	0.001 (3)	−0.041 (4)
F3	0.0203 (17)	0.0193 (14)	0.0465 (18)	0.0022 (12)	−0.0024 (14)	−0.0060 (12)
F4	0.0327 (18)	0.0242 (14)	0.0307 (15)	−0.0107 (13)	−0.0047 (13)	−0.0084 (12)
O1	0.020 (2)	0.0295 (18)	0.0294 (18)	−0.0095 (15)	−0.0020 (15)	−0.0104 (14)
O2	0.023 (2)	0.0186 (16)	0.0300 (18)	−0.0023 (15)	0.0043 (15)	−0.0004 (14)
O3	0.0178 (18)	0.0216 (16)	0.0220 (16)	−0.0112 (14)	−0.0002 (14)	−0.0012 (13)
O4	0.0201 (19)	0.0148 (15)	0.0239 (17)	−0.0009 (14)	−0.0021 (14)	0.0031 (13)
C1	0.021 (3)	0.017 (2)	0.019 (2)	−0.013 (2)	0.009 (2)	−0.0082 (18)
C2	0.023 (3)	0.033 (3)	0.044 (3)	−0.007 (2)	−0.001 (3)	−0.021 (2)
C3	0.007 (2)	0.016 (2)	0.017 (2)	−0.0036 (18)	−0.0012 (17)	−0.0001 (16)
C4	0.020 (3)	0.019 (2)	0.015 (2)	−0.006 (2)	−0.0015 (19)	0.0017 (17)
C5	0.022 (3)	0.017 (2)	0.022 (2)	−0.005 (2)	−0.002 (2)	−0.0024 (18)
C6	0.014 (3)	0.019 (2)	0.024 (2)	−0.0048 (19)	0.0042 (19)	0.0008 (18)
C7	0.016 (3)	0.021 (2)	0.019 (2)	−0.004 (2)	−0.0037 (19)	0.0054 (18)
C8	0.012 (2)	0.021 (2)	0.018 (2)	−0.0034 (19)	−0.0001 (18)	0.0004 (17)
C9	0.016 (2)	0.012 (2)	0.016 (2)	−0.0035 (18)	−0.0012 (18)	−0.0003 (16)
C10	0.015 (3)	0.020 (2)	0.021 (2)	−0.003 (2)	0.0019 (19)	−0.0012 (18)
C11	0.025 (3)	0.018 (2)	0.027 (3)	−0.002 (2)	0.009 (2)	0.0006 (19)
C12	0.039 (3)	0.021 (2)	0.024 (3)	−0.007 (2)	0.001 (2)	−0.008 (2)
C13	0.034 (3)	0.021 (2)	0.024 (2)	−0.015 (2)	0.001 (2)	−0.0048 (19)
C14	0.020 (3)	0.022 (2)	0.018 (2)	−0.008 (2)	−0.0010 (19)	−0.0002 (18)
C15	0.017 (2)	0.013 (2)	0.015 (2)	−0.0049 (18)	−0.0005 (18)	−0.0024 (16)
C16	0.014 (2)	0.024 (2)	0.011 (2)	−0.0063 (19)	0.0009 (18)	−0.0048 (17)
C17	0.021 (3)	0.026 (2)	0.018 (2)	−0.009 (2)	−0.004 (2)	0.0018 (18)
C18	0.016 (3)	0.044 (3)	0.024 (3)	−0.008 (2)	0.004 (2)	−0.007 (2)
C19	0.022 (3)	0.045 (3)	0.032 (3)	−0.003 (3)	0.004 (2)	−0.024 (2)
C20	0.019 (3)	0.029 (3)	0.045 (3)	0.000 (2)	−0.004 (2)	−0.020 (2)
C21	0.024 (3)	0.027 (3)	0.031 (3)	−0.012 (2)	0.001 (2)	−0.009 (2)
C22	0.016 (2)	0.014 (2)	0.013 (2)	−0.0093 (18)	0.0002 (18)	0.0012 (16)
C23	0.010 (2)	0.015 (2)	0.020 (2)	−0.0039 (18)	0.0005 (18)	0.0000 (17)
C24	0.014 (3)	0.023 (2)	0.022 (2)	−0.010 (2)	−0.0038 (19)	0.0060 (18)
C25	0.019 (3)	0.030 (3)	0.014 (2)	−0.012 (2)	−0.0054 (19)	0.0037 (18)
C26	0.028 (3)	0.025 (2)	0.019 (2)	−0.014 (2)	0.004 (2)	−0.0053 (19)
C27	0.016 (3)	0.017 (2)	0.021 (2)	−0.0079 (19)	−0.0008 (19)	0.0000 (17)
C28	0.015 (3)	0.012 (2)	0.021 (2)	−0.0068 (19)	0.0046 (19)	−0.0003 (17)
C29	0.016 (3)	0.015 (2)	0.024 (2)	−0.0062 (19)	−0.0003 (19)	−0.0011 (17)
C30	0.016 (2)	0.013 (2)	0.014 (2)	−0.0036 (18)	0.0009 (18)	−0.0013 (16)
C31	0.011 (2)	0.021 (2)	0.013 (2)	−0.0050 (19)	−0.0018 (17)	−0.0012 (17)
C32	0.020 (3)	0.012 (2)	0.020 (2)	0.0007 (19)	−0.004 (2)	−0.0034 (17)
C33	0.020 (3)	0.019 (2)	0.023 (2)	0.004 (2)	0.002 (2)	−0.0086 (19)
C34	0.013 (3)	0.031 (3)	0.023 (2)	−0.003 (2)	0.001 (2)	−0.006 (2)
C35	0.018 (3)	0.021 (2)	0.020 (2)	−0.009 (2)	0.0002 (19)	−0.0050 (18)
C36	0.013 (2)	0.015 (2)	0.014 (2)	−0.0087 (18)	0.0016 (17)	−0.0031 (16)
C37	0.013 (2)	0.017 (2)	0.015 (2)	−0.0030 (19)	0.0003 (18)	−0.0002 (17)
C38	0.019 (3)	0.038 (3)	0.016 (2)	−0.009 (2)	−0.001 (2)	0.001 (2)
C39	0.022 (3)	0.038 (3)	0.018 (2)	−0.021 (2)	0.002 (2)	−0.007 (2)
C40	0.030 (3)	0.019 (2)	0.027 (2)	−0.016 (2)	0.004 (2)	−0.0079 (19)
C41	0.023 (3)	0.018 (2)	0.018 (2)	−0.006 (2)	−0.002 (2)	0.0007 (17)
C42	0.015 (2)	0.014 (2)	0.013 (2)	−0.0052 (18)	−0.0007 (17)	−0.0006 (16)
C43	0.018 (3)	0.0097 (19)	0.013 (2)	−0.0049 (18)	−0.0027 (18)	−0.0007 (15)
C44	0.019 (3)	0.022 (2)	0.019 (2)	−0.007 (2)	0.0006 (19)	−0.0004 (18)
C45	0.026 (3)	0.025 (3)	0.028 (3)	−0.001 (2)	0.003 (2)	−0.010 (2)
C46	0.047 (4)	0.016 (2)	0.030 (3)	−0.007 (2)	0.000 (3)	−0.009 (2)
C47	0.050 (4)	0.025 (3)	0.026 (3)	−0.024 (3)	0.001 (3)	−0.005 (2)
C48	0.024 (3)	0.023 (2)	0.022 (2)	−0.012 (2)	0.002 (2)	−0.0037 (19)
C49	0.007 (2)	0.017 (2)	0.013 (2)	−0.0013 (18)	0.0032 (17)	0.0004 (16)
C50	0.011 (2)	0.019 (2)	0.018 (2)	−0.0071 (19)	0.0013 (18)	−0.0008 (17)
C51	0.011 (2)	0.014 (2)	0.026 (2)	−0.0019 (18)	0.0048 (19)	0.0013 (18)
C52	0.021 (3)	0.023 (2)	0.020 (2)	−0.006 (2)	−0.003 (2)	0.0061 (18)
C53	0.022 (3)	0.028 (3)	0.016 (2)	−0.008 (2)	−0.001 (2)	−0.0031 (19)
C54	0.020 (3)	0.014 (2)	0.018 (2)	−0.0048 (19)	0.0010 (19)	−0.0026 (17)

### Geometric parameters (Å, °)

**Table:**

Ag1—O1	2.382 (3)	C18—H18	0.9500
Ag1—P1	2.4194 (12)	C19—C20	1.370 (7)
Ag1—P2^i^	2.419 (1)	C19—H19	0.9500
Ag1—Ag1^i^	3.1078 (6)	C20—C21	1.402 (7)
Ag2—O3	2.383 (3)	C20—H20	0.9500
Ag2—P3	2.430 (1)	C21—H21	0.9500
Ag2—P4^ii^	2.429 (1)	C22—C23	1.392 (6)
Ag2—Ag2^ii^	2.9950 (6)	C22—C27	1.401 (6)
P1—C9	1.810 (4)	C23—C24	1.395 (6)
P1—C3	1.821 (4)	C23—H23	0.9500
P1—C15	1.834 (4)	C24—C25	1.376 (6)
P2—C16	1.813 (4)	C24—H24	0.9500
P2—C22	1.822 (4)	C25—C26	1.385 (6)
P2—C15	1.836 (4)	C25—H25	0.9500
P2—Ag1^i^	2.4188 (12)	C26—C27	1.370 (6)
P3—C30	1.816 (4)	C26—H26	0.9500
P3—C36	1.825 (4)	C27—H27	0.9500
P3—C42	1.843 (4)	C28—C29	1.539 (5)
P4—C43	1.816 (4)	C30—C35	1.387 (6)
P4—C49	1.818 (4)	C30—C31	1.397 (6)
P4—C42	1.834 (4)	C31—C32	1.395 (6)
P4—Ag2^ii^	2.4291 (12)	C31—H31	0.9500
Cl1—C2	1.683 (5)	C32—C33	1.380 (7)
Cl1'—C2	1.652 (5)	C32—H32	0.9500
Cl2—C29	1.737 (4)	C33—C34	1.387 (6)
F1—C2	1.413 (6)	C33—H33	0.9500
F2—C2	1.375 (7)	C34—C35	1.395 (6)
F1'—C2	1.402 (6)	C34—H34	0.9500
F2'—C2	1.378 (7)	C35—H35	0.9500
F3—C29	1.360 (4)	C36—C37	1.390 (6)
F4—C29	1.354 (4)	C36—C41	1.392 (6)
O1—C1	1.235 (5)	C37—C38	1.392 (6)
O2—C1	1.236 (5)	C37—H37	0.9500
O3—C28	1.254 (5)	C38—C39	1.378 (7)
O4—C28	1.240 (5)	C38—H38	0.9500
C1—C2	1.547 (6)	C39—C40	1.377 (7)
C3—C4	1.392 (6)	C39—H39	0.9500
C3—C8	1.397 (5)	C40—C41	1.382 (6)
C4—C5	1.372 (6)	C40—H40	0.9500
C4—H4	0.9500	C41—H41	0.9500
C5—C6	1.388 (6)	C42—H42A	0.9900
C5—H5	0.9500	C42—H42B	0.9900
C6—C7	1.389 (6)	C43—C44	1.388 (6)
C6—H6	0.9500	C43—C48	1.397 (6)
C7—C8	1.386 (6)	C44—C45	1.376 (6)
C7—H7	0.9500	C44—H44	0.9500
C8—H8	0.9500	C45—C46	1.402 (7)
C9—C14	1.386 (6)	C45—H45	0.9500
C9—C10	1.396 (6)	C46—C47	1.376 (8)
C10—C11	1.390 (6)	C46—H46	0.9500
C10—H10	0.9500	C47—C48	1.395 (6)
C11—C12	1.393 (7)	C47—H47	0.9500
C11—H11	0.9500	C48—H48	0.9500
C12—C13	1.373 (7)	C49—C50	1.387 (6)
C12—H12	0.9500	C49—C54	1.396 (6)
C13—C14	1.378 (6)	C50—C51	1.387 (6)
C13—H13	0.9500	C50—H50	0.9500
C14—H14	0.9500	C51—C52	1.380 (6)
C15—H15A	0.9900	C51—H51	0.9500
C15—H15B	0.9900	C52—C53	1.378 (6)
C16—C17	1.390 (6)	C52—H52	0.9500
C16—C21	1.397 (6)	C53—C54	1.387 (6)
C17—C18	1.384 (7)	C53—H53	0.9500
C17—H17	0.9500	C54—H54	0.9500
C18—C19	1.392 (7)		
			
O1—Ag1—P1	100.92 (9)	C21—C20—H20	119.9
O1—Ag1—P2^i^	103.78 (9)	C16—C21—C20	119.4 (5)
P1—Ag1—P2^i^	151.19 (4)	C16—C21—H21	120.3
O1—Ag1—Ag1^i^	136.10 (8)	C20—C21—H21	120.3
P2^i^—Ag1—Ag1^i^	91.48 (3)	C23—C22—C27	118.9 (4)
P1—Ag1—Ag1^i^	80.72 (3)	C23—C22—P2	122.4 (3)
O3—Ag2—P3	104.14 (8)	C27—C22—P2	118.4 (3)
O3—Ag2—P4^ii^	101.43 (8)	C22—C23—C24	120.1 (4)
P3—Ag2—P4^ii^	149.67 (4)	C22—C23—H23	120.0
O3—Ag2—Ag2^ii^	134.22 (7)	C24—C23—H23	120.0
P4^ii^—Ag2—Ag2^ii^	78.04 (3)	C25—C24—C23	120.0 (4)
P3—Ag2—Ag2^ii^	95.23 (3)	C25—C24—H24	120.0
C9—P1—C3	105.99 (19)	C23—C24—H24	120.0
C9—P1—C15	103.98 (19)	C24—C25—C26	120.1 (4)
C3—P1—C15	102.13 (19)	C24—C25—H25	119.9
C9—P1—Ag1	119.10 (15)	C26—C25—H25	119.9
C3—P1—Ag1	113.66 (15)	C27—C26—C25	120.4 (4)
C15—P1—Ag1	110.26 (15)	C27—C26—H26	119.8
C16—P2—C22	103.7 (2)	C25—C26—H26	119.8
C16—P2—C15	106.9 (2)	C26—C27—C22	120.5 (4)
C22—P2—C15	104.17 (18)	C26—C27—H27	119.8
C16—P2—Ag1^i^	118.83 (15)	C22—C27—H27	119.8
C22—P2—Ag1^i^	109.27 (15)	O4—C28—O3	129.2 (4)
C15—P2—Ag1^i^	112.61 (15)	O4—C28—C29	115.7 (4)
C30—P3—C36	105.1 (2)	O3—C28—C29	115.0 (4)
C30—P3—C42	105.46 (19)	F4—C29—F3	104.5 (3)
C36—P3—C42	104.87 (18)	F4—C29—C28	111.2 (3)
C30—P3—Ag2	119.03 (15)	F3—C29—C28	111.3 (3)
C36—P3—Ag2	112.02 (15)	F4—C29—Cl2	108.8 (3)
C42—P3—Ag2	109.25 (15)	F3—C29—Cl2	109.3 (3)
C43—P4—C49	104.85 (19)	C28—C29—Cl2	111.5 (3)
C43—P4—C42	102.48 (19)	C35—C30—C31	119.7 (4)
C49—P4—C42	105.27 (19)	C35—C30—P3	121.1 (3)
C43—P4—Ag2^ii^	121.28 (15)	C31—C30—P3	119.3 (3)
C49—P4—Ag2^ii^	109.90 (14)	C32—C31—C30	119.2 (4)
C42—P4—Ag2^ii^	111.71 (14)	C32—C31—H31	120.4
C1—O1—Ag1	107.5 (3)	C30—C31—H31	120.4
C28—O3—Ag2	109.5 (3)	C33—C32—C31	120.7 (4)
O1—C1—O2	130.1 (4)	C33—C32—H32	119.7
O1—C1—C2	113.9 (4)	C31—C32—H32	119.7
O2—C1—C2	115.9 (4)	C32—C33—C34	120.5 (4)
F2'—C2—F1'	100.4 (6)	C32—C33—H33	119.8
F2—C2—F1	99.3 (6)	C34—C33—H33	119.8
F2—C2—C1	113.2 (7)	C33—C34—C35	119.1 (5)
F2'—C2—C1	108.0 (8)	C33—C34—H34	120.5
F1'—C2—C1	109.5 (5)	C35—C34—H34	120.5
F1—C2—C1	105.0 (5)	C30—C35—C34	120.9 (4)
F2'—C2—Cl1'	111.8 (6)	C30—C35—H35	119.6
F1'—C2—Cl1'	109.7 (4)	C34—C35—H35	119.6
C1—C2—Cl1'	116.3 (3)	C37—C36—C41	118.4 (4)
F2—C2—Cl1	111.4 (6)	C37—C36—P3	123.0 (3)
F1—C2—Cl1	106.8 (5)	C41—C36—P3	118.2 (3)
C1—C2—Cl1	118.8 (3)	C36—C37—C38	120.5 (4)
C4—C3—C8	119.5 (4)	C36—C37—H37	119.7
C4—C3—P1	119.6 (3)	C38—C37—H37	119.7
C8—C3—P1	120.8 (3)	C39—C38—C37	119.8 (4)
C5—C4—C3	120.8 (4)	C39—C38—H38	120.1
C5—C4—H4	119.6	C37—C38—H38	120.1
C3—C4—H4	119.6	C40—C39—C38	120.6 (4)
C4—C5—C6	120.3 (4)	C40—C39—H39	119.7
C4—C5—H5	119.9	C38—C39—H39	119.7
C6—C5—H5	119.9	C39—C40—C41	119.6 (4)
C5—C6—C7	119.2 (4)	C39—C40—H40	120.2
C5—C6—H6	120.4	C41—C40—H40	120.2
C7—C6—H6	120.4	C40—C41—C36	121.2 (4)
C8—C7—C6	121.1 (4)	C40—C41—H41	119.4
C8—C7—H7	119.5	C36—C41—H41	119.4
C6—C7—H7	119.5	P4—C42—P3	108.4 (2)
C7—C8—C3	119.1 (4)	P4—C42—H42A	110.0
C7—C8—H8	120.4	P3—C42—H42A	110.0
C3—C8—H8	120.4	P4—C42—H42B	110.0
C14—C9—C10	119.2 (4)	P3—C42—H42B	110.0
C14—C9—P1	119.6 (4)	H42A—C42—H42B	108.4
C10—C9—P1	121.2 (3)	C44—C43—C48	119.6 (4)
C11—C10—C9	119.7 (4)	C44—C43—P4	120.6 (3)
C11—C10—H10	120.1	C48—C43—P4	119.7 (4)
C9—C10—H10	120.1	C45—C44—C43	120.8 (4)
C10—C11—C12	120.2 (5)	C45—C44—H44	119.6
C10—C11—H11	119.9	C43—C44—H44	119.6
C12—C11—H11	119.9	C44—C45—C46	119.6 (5)
C13—C12—C11	119.8 (4)	C44—C45—H45	120.2
C13—C12—H12	120.1	C46—C45—H45	120.2
C11—C12—H12	120.1	C47—C46—C45	119.9 (4)
C12—C13—C14	120.4 (5)	C47—C46—H46	120.0
C12—C13—H13	119.8	C45—C46—H46	120.0
C14—C13—H13	119.8	C46—C47—C48	120.5 (5)
C13—C14—C9	120.8 (5)	C46—C47—H47	119.8
C13—C14—H14	119.6	C48—C47—H47	119.8
C9—C14—H14	119.6	C47—C48—C43	119.5 (5)
P1—C15—P2	110.3 (2)	C47—C48—H48	120.3
P1—C15—H15A	109.6	C43—C48—H48	120.3
P2—C15—H15A	109.6	C50—C49—C54	118.8 (4)
P1—C15—H15B	109.6	C50—C49—P4	120.2 (3)
P2—C15—H15B	109.6	C54—C49—P4	120.7 (3)
H15A—C15—H15B	108.1	C51—C50—C49	120.7 (4)
C17—C16—C21	119.4 (4)	C51—C50—H50	119.7
C17—C16—P2	122.7 (3)	C49—C50—H50	119.7
C21—C16—P2	117.2 (4)	C52—C51—C50	119.9 (4)
C18—C17—C16	120.9 (5)	C52—C51—H51	120.0
C18—C17—H17	119.5	C50—C51—H51	120.0
C16—C17—H17	119.5	C53—C52—C51	120.1 (4)
C17—C18—C19	119.2 (5)	C53—C52—H52	120.0
C17—C18—H18	120.4	C51—C52—H52	120.0
C19—C18—H18	120.4	C52—C53—C54	120.2 (4)
C20—C19—C18	120.8 (5)	C52—C53—H53	119.9
C20—C19—H19	119.6	C54—C53—H53	119.9
C18—C19—H19	119.6	C53—C54—C49	120.2 (4)
C19—C20—C21	120.2 (5)	C53—C54—H54	119.9
C19—C20—H20	119.9	C49—C54—H54	119.9
			
O1—Ag1—P1—C9	158.28 (17)	C19—C20—C21—C16	1.4 (7)
P2^i^—Ag1—P1—C9	9.62 (18)	C16—P2—C22—C23	14.1 (4)
Ag1^i^—Ag1—P1—C9	−66.32 (15)	C15—P2—C22—C23	125.9 (4)
O1—Ag1—P1—C3	32.26 (17)	Ag1^i^—P2—C22—C23	−113.5 (3)
P2^i^—Ag1—P1—C3	−116.40 (16)	C16—P2—C22—C27	−172.3 (3)
Ag1^i^—Ag1—P1—C3	167.66 (15)	C15—P2—C22—C27	−60.5 (4)
O1—Ag1—P1—C15	−81.72 (16)	Ag1^i^—P2—C22—C27	60.0 (3)
P2^i^—Ag1—P1—C15	129.63 (15)	C27—C22—C23—C24	−0.3 (6)
Ag1^i^—Ag1—P1—C15	53.68 (14)	P2—C22—C23—C24	173.3 (3)
O3—Ag2—P3—C30	−11.10 (17)	C22—C23—C24—C25	0.1 (6)
P4^ii^—Ag2—P3—C30	−157.73 (15)	C23—C24—C25—C26	0.3 (7)
Ag2^ii^—Ag2—P3—C30	127.08 (15)	C24—C25—C26—C27	−0.5 (7)
O3—Ag2—P3—C36	112.04 (16)	C25—C26—C27—C22	0.3 (7)
P4^ii^—Ag2—P3—C36	−34.59 (17)	C23—C22—C27—C26	0.1 (6)
Ag2^ii^—Ag2—P3—C36	−109.78 (14)	P2—C22—C27—C26	−173.7 (3)
O3—Ag2—P3—C42	−132.21 (16)	Ag2—O3—C28—O4	11.0 (6)
P4^ii^—Ag2—P3—C42	81.16 (16)	Ag2—O3—C28—C29	−172.5 (3)
Ag2^ii^—Ag2—P3—C42	5.97 (14)	O4—C28—C29—F4	−29.1 (5)
P2^i^—Ag1—O1—C1	−129.4 (3)	O3—C28—C29—F4	153.9 (4)
P1—Ag1—O1—C1	65.6 (3)	O4—C28—C29—F3	−145.2 (4)
Ag1^i^—Ag1—O1—C1	−22.3 (4)	O3—C28—C29—F3	37.9 (5)
P4^ii^—Ag2—O3—C28	−65.3 (3)	O4—C28—C29—Cl2	92.5 (4)
P3—Ag2—O3—C28	131.1 (3)	O3—C28—C29—Cl2	−84.4 (4)
Ag2^ii^—Ag2—O3—C28	19.0 (3)	C36—P3—C30—C35	60.9 (4)
Ag1—O1—C1—O2	−3.4 (6)	C42—P3—C30—C35	−49.6 (4)
Ag1—O1—C1—C2	178.4 (3)	Ag2—P3—C30—C35	−172.6 (3)
O1—C1—C2—F2	−18.6 (7)	C36—P3—C30—C31	−118.2 (3)
O2—C1—C2—F2	163.0 (6)	C42—P3—C30—C31	131.3 (3)
O1—C1—C2—F2'	−39.4 (7)	Ag2—P3—C30—C31	8.2 (4)
O2—C1—C2—F2'	142.2 (6)	C35—C30—C31—C32	0.5 (6)
O1—C1—C2—F1'	−147.8 (6)	P3—C30—C31—C32	179.7 (3)
O2—C1—C2—F1'	33.8 (7)	C30—C31—C32—C33	−0.5 (6)
O1—C1—C2—F1	88.6 (6)	C31—C32—C33—C34	0.2 (7)
O2—C1—C2—F1	−89.8 (6)	C32—C33—C34—C35	0.2 (7)
O1—C1—C2—Cl1'	87.2 (5)	C31—C30—C35—C34	−0.1 (6)
O2—C1—C2—Cl1'	−91.3 (5)	P3—C30—C35—C34	−179.2 (3)
O1—C1—C2—Cl1	−152.1 (4)	C33—C34—C35—C30	−0.3 (7)
O2—C1—C2—Cl1	29.5 (6)	C30—P3—C36—C37	17.4 (4)
C9—P1—C3—C4	150.5 (4)	C42—P3—C36—C37	128.4 (4)
C15—P1—C3—C4	41.9 (4)	Ag2—P3—C36—C37	−113.2 (4)
Ag1—P1—C3—C4	−76.9 (4)	C30—P3—C36—C41	−169.0 (3)
C9—P1—C3—C8	−33.6 (4)	C42—P3—C36—C41	−58.0 (4)
C15—P1—C3—C8	−142.2 (4)	Ag2—P3—C36—C41	60.3 (4)
Ag1—P1—C3—C8	99.1 (4)	C41—C36—C37—C38	−0.5 (7)
C8—C3—C4—C5	1.6 (7)	P3—C36—C37—C38	173.0 (4)
P1—C3—C4—C5	177.5 (4)	C36—C37—C38—C39	0.6 (7)
C3—C4—C5—C6	−0.3 (7)	C37—C38—C39—C40	0.1 (7)
C4—C5—C6—C7	−0.6 (7)	C38—C39—C40—C41	−0.7 (7)
C5—C6—C7—C8	0.2 (7)	C39—C40—C41—C36	0.7 (7)
C6—C7—C8—C3	1.0 (7)	C37—C36—C41—C40	−0.1 (7)
C4—C3—C8—C7	−1.9 (7)	P3—C36—C41—C40	−174.0 (4)
P1—C3—C8—C7	−177.8 (3)	C43—P4—C42—P3	63.2 (3)
C3—P1—C9—C14	142.2 (3)	C49—P4—C42—P3	172.6 (2)
C15—P1—C9—C14	−110.6 (4)	Ag2^ii^—P4—C42—P3	−68.2 (2)
Ag1—P1—C9—C14	12.6 (4)	C30—P3—C42—P4	−97.8 (2)
C3—P1—C9—C10	−40.2 (4)	C36—P3—C42—P4	151.5 (2)
C15—P1—C9—C10	67.0 (4)	Ag2—P3—C42—P4	31.2 (2)
Ag1—P1—C9—C10	−169.8 (3)	C49—P4—C43—C44	−54.0 (4)
C14—C9—C10—C11	−1.5 (6)	C42—P4—C43—C44	55.8 (4)
P1—C9—C10—C11	−179.1 (3)	Ag2^ii^—P4—C43—C44	−178.9 (3)
C9—C10—C11—C12	1.3 (7)	C49—P4—C43—C48	129.3 (3)
C10—C11—C12—C13	0.2 (7)	C42—P4—C43—C48	−121.0 (3)
C11—C12—C13—C14	−1.5 (7)	Ag2^ii^—P4—C43—C48	4.3 (4)
C12—C13—C14—C9	1.3 (7)	C48—C43—C44—C45	0.8 (6)
C10—C9—C14—C13	0.2 (6)	P4—C43—C44—C45	−176.0 (3)
P1—C9—C14—C13	177.9 (3)	C43—C44—C45—C46	1.1 (7)
C9—P1—C15—P2	62.7 (3)	C44—C45—C46—C47	−1.9 (7)
C3—P1—C15—P2	172.8 (2)	C45—C46—C47—C48	0.7 (7)
Ag1—P1—C15—P2	−66.1 (2)	C46—C47—C48—C43	1.2 (7)
C16—P2—C15—P1	−99.2 (2)	C44—C43—C48—C47	−2.0 (6)
C22—P2—C15—P1	151.4 (2)	P4—C43—C48—C47	174.8 (3)
Ag1^i^—P2—C15—P1	33.1 (2)	C43—P4—C49—C50	155.4 (4)
C22—P2—C16—C17	75.3 (4)	C42—P4—C49—C50	47.7 (4)
C15—P2—C16—C17	−34.4 (4)	Ag2^ii^—P4—C49—C50	−72.7 (4)
Ag1^i^—P2—C16—C17	−163.2 (3)	C43—P4—C49—C54	−31.0 (4)
C22—P2—C16—C21	−95.5 (4)	C42—P4—C49—C54	−138.7 (4)
C15—P2—C16—C21	154.8 (3)	Ag2^ii^—P4—C49—C54	100.9 (3)
Ag1^i^—P2—C16—C21	26.0 (4)	C54—C49—C50—C51	1.2 (7)
C21—C16—C17—C18	−0.9 (7)	P4—C49—C50—C51	174.9 (3)
P2—C16—C17—C18	−171.5 (3)	C49—C50—C51—C52	−0.5 (7)
C16—C17—C18—C19	1.8 (7)	C50—C51—C52—C53	−1.0 (7)
C17—C18—C19—C20	−1.1 (7)	C51—C52—C53—C54	1.7 (7)
C18—C19—C20—C21	−0.5 (8)	C52—C53—C54—C49	−1.0 (7)
C17—C16—C21—C20	−0.7 (7)	C50—C49—C54—C53	−0.5 (7)
P2—C16—C21—C20	170.4 (4)	P4—C49—C54—C53	−174.1 (4)

Symmetry codes: (i) −*x*+1, −*y*+2, −*z*+2; (ii) −*x*+1, −*y*+1, −*z*+1.
